# 

**Published:** 1895-04

**Authors:** 


### CONTENTS—APRIL, 1895.—VOL. X., NO. 10.

ORIGINAL COMMUNICATIONS—

Hemorrhoids and Treatment. C. T. Young, M. D., Waco . . . . 505
Metritis. Robert T. Morris, M. D., Houston. . . . . . . . . . . . 514
Hip Disease in Children. Dr. Samuel E. Milliken, New York . . . 520

SOCIETY NOTES—

Asylum in Politics—Medical Legislation—Medical Organization, etc. Daniel Parker . . . . . . . . . . . . . . . . . . . . . . . . . 522

SOCIETY NOTES—

Preliminary Announcement and Program State Medical Association 526
A Medico-Legal Congress . . . . . . . . . . . . . . . . . . . . 531

EDITORIAL—

The Legislature and the Doctors . . . . . . . . . . . . . . . . . 533
Relationship of National and State Quarantine . . . . . . . . . . 535
The Pervert Wilde . . . . . . . . . . . . . . . . . . . . . . . . 536
Governor Clark and the Practice of Medicine . . . . . . . . . . . 538
Ho! for Baltimore!. . . . . . . . . . . . . . . . . . . . . . . . 540
Meeting of the Texas State Medical Association . . . . . . . . . . 540
Pacific Coast Association of Examiners . . . . . . . . . . . . . 541
American Medical Association Meeting at Baltimore . . . . . . . . 542

MEDICAL NEWS AND MISCELLANY—

Many interesting paragraphs . . . . . . . . . . . . . . . . . . .
The Antitoxine Treatment of Diphtheria . . . . . . . . . . . . . 555
Hydrophobia Inoculation. . . . . . . . . . . . . . . . . . . . . . 556

BOOK NOTICES— . . . . . . . . . . . . . . . . . . . . . . . . . . 557

PUBLISHERS' NOTES . . . . . . . . . . . . . . . . . . . . . . . . 563

